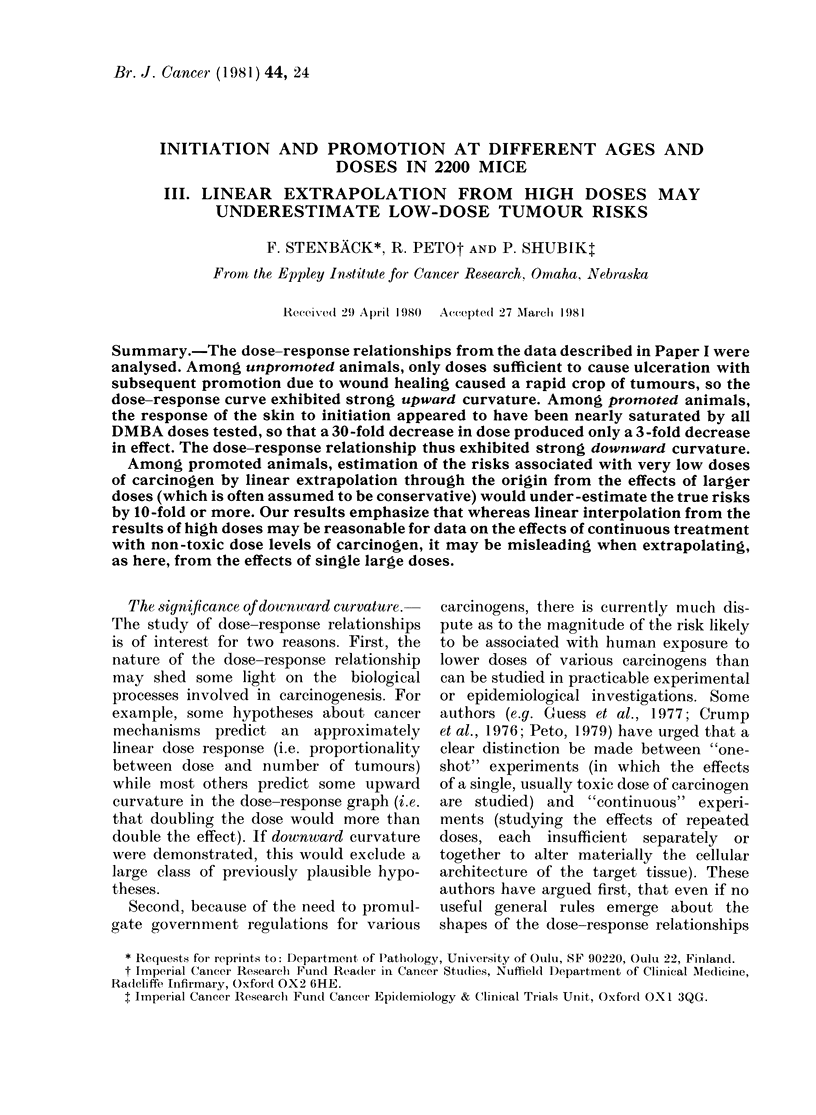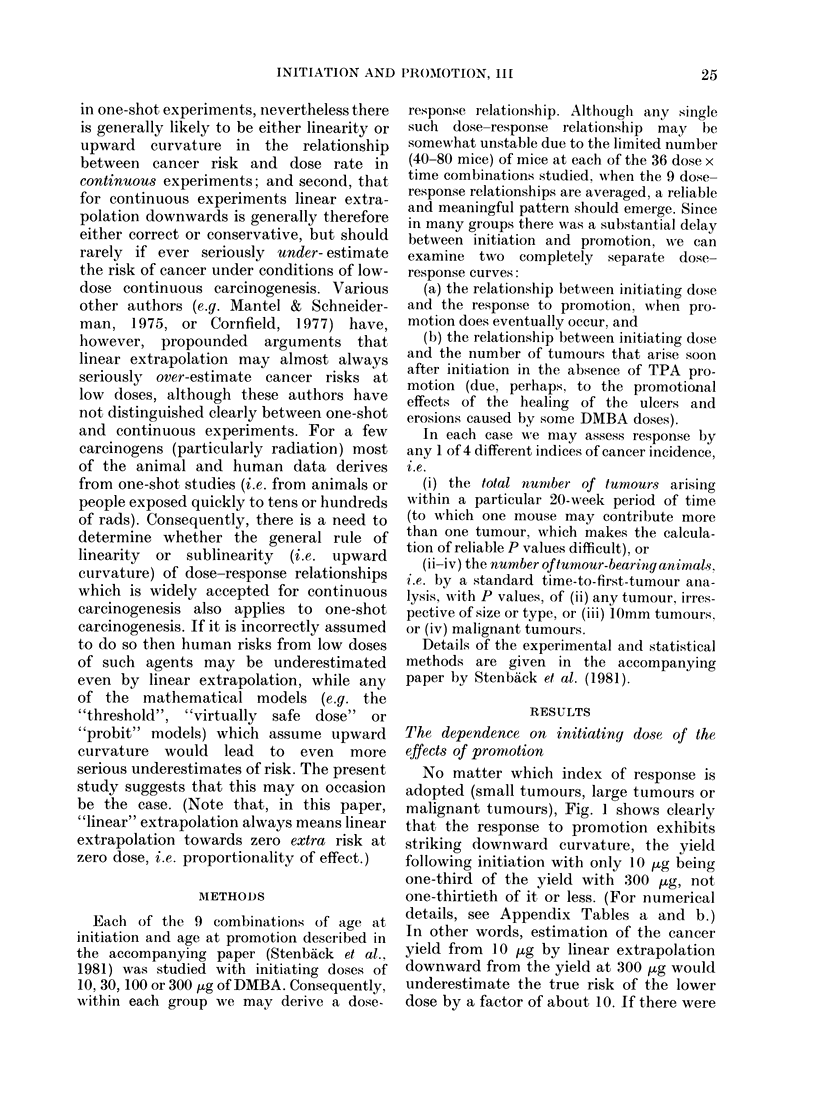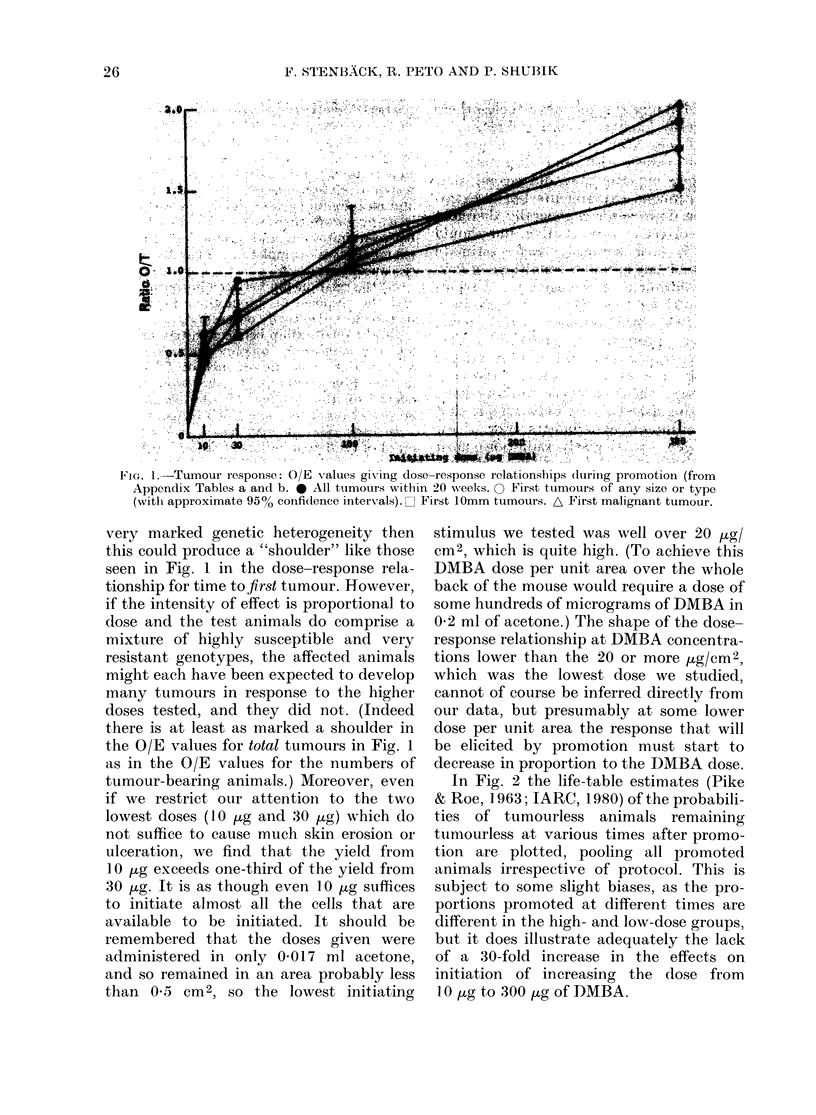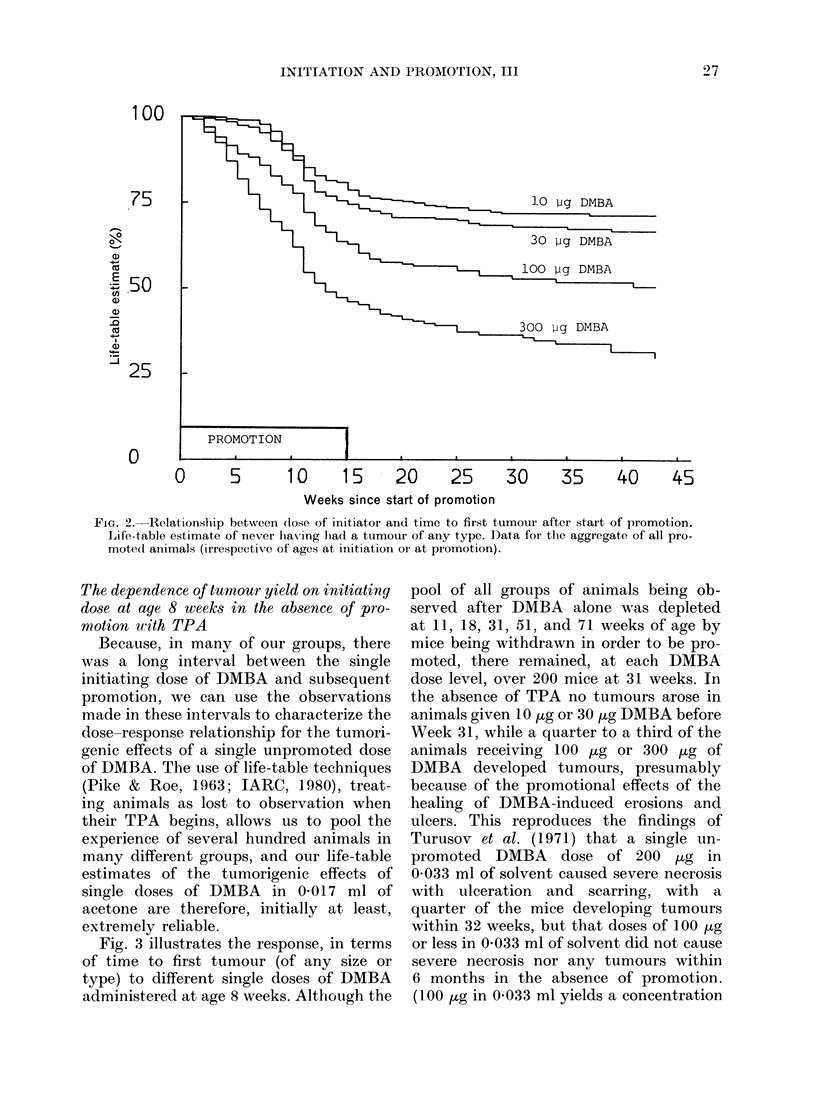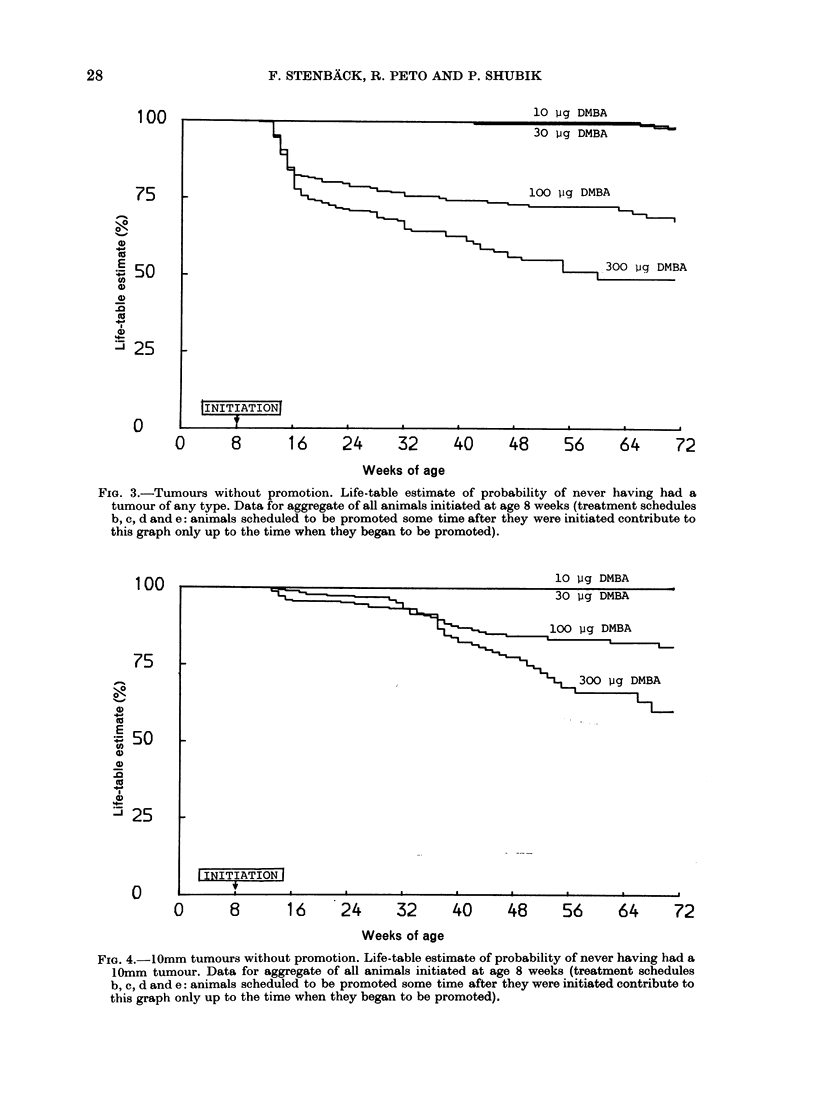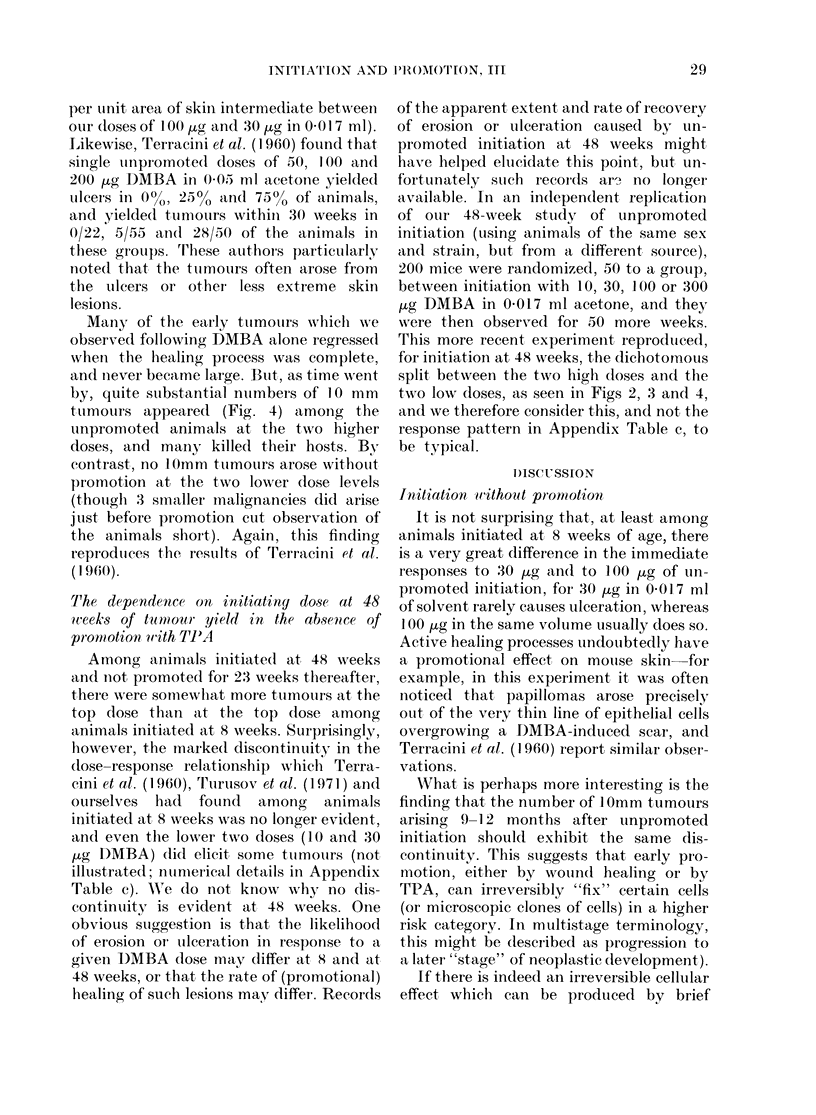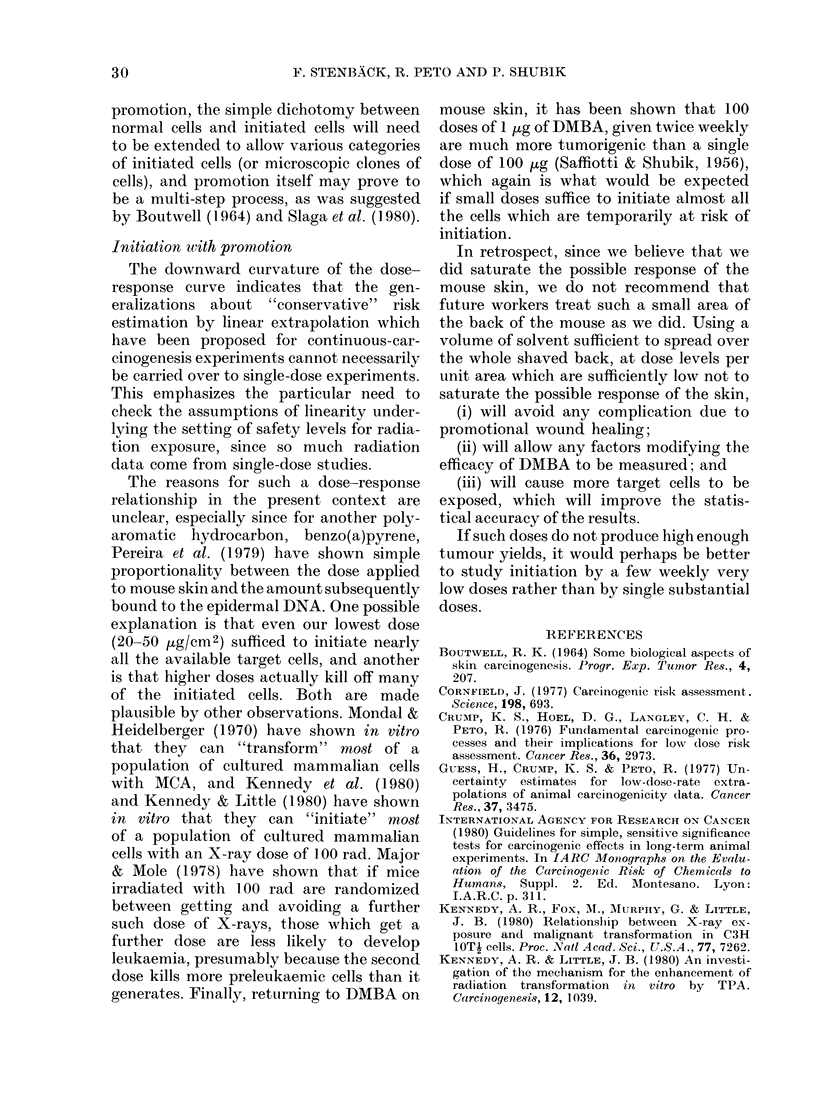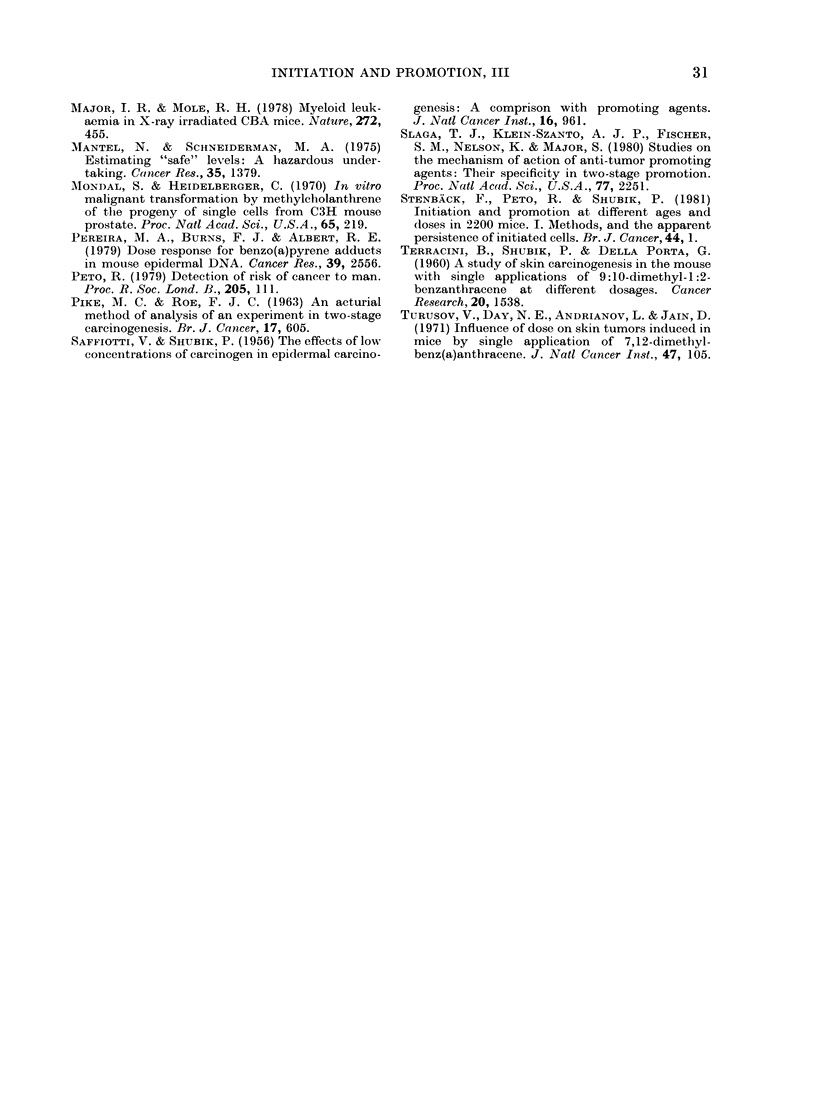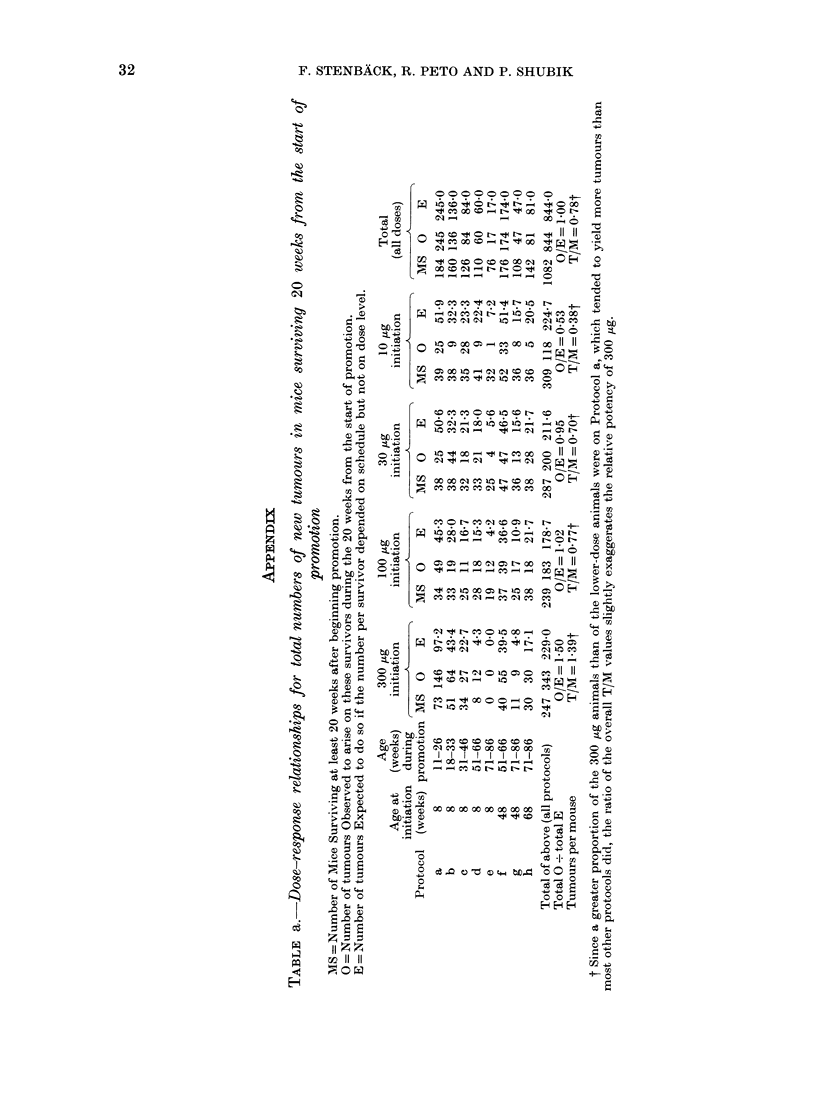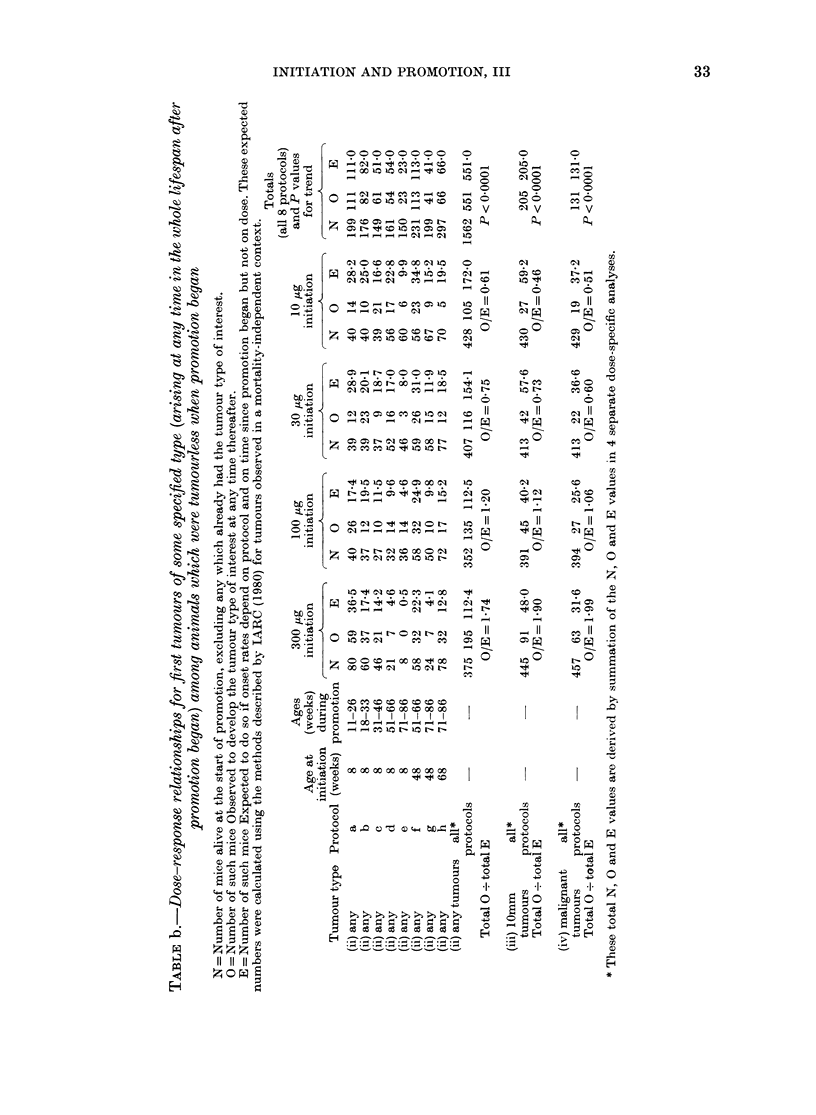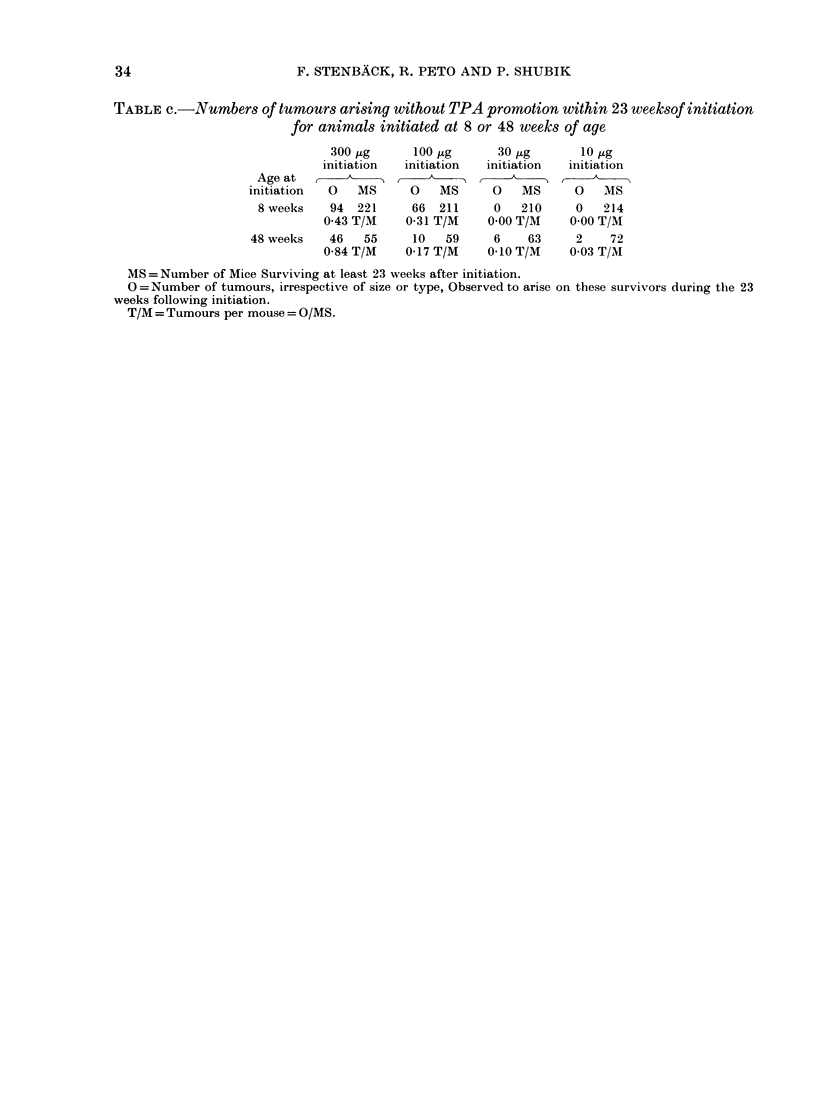# Initiation and promotion at different ages and doses in 2200 mice. III. Linear extrapolation from high doses may underestimate low-dose tumour risks.

**DOI:** 10.1038/bjc.1981.143

**Published:** 1981-07

**Authors:** F. Stenbäck, R. Peto, P. Shubik

## Abstract

The dose-response relationships from the data described in Paper I were analysed. Among unpromoted animals, only doses sufficient to cause ulceration with subsequent promotion due to wound healing caused a rapid crop of tumours, so the dose-response curve exhibited strong upward curvature. Among promoted animals, the response of the skin to initiation appeared to have been nearly saturated by all DMBA doses tested, so that a 30-fold decrease in dose produced only a 3-fold decrease in effect. The dose-response relationship thus exhibited strong downward curvature. Among promoted animals, estimation of the risks associated with very low doses of carcinogen by linear extrapolation through the origin from the effects of larger doses (which is often assumed to be conservative) would under-estimate the true risks by 10-fold or more. Our results emphasize that whereas linear interpolation from the results of high doses may be reasonable for data on the effects of continuous treatment with non-toxic dose levels of carcinogen, it may be misleading when extrapolating, as here, from the effects of single large doses.


*Br. J. Cancer* (1981) **44**, 24

### INITIATION AND PROMOTION AT DIFFERENT AGES AND DOSES IN 2200 MICE

### III. LINEAR EXTRAPOLATION FROM HIGH DOSES MAY UNDERESTIMATE LOW-DOSE TUMOUR RISKS

F. STENBÄCK*, R. PETO† AND P. SHUBIK‡

*From the Eppley Institute for Cancer Research, Omaha, Nebraska*

Received 29 April 1980 Accepted 27 March 1981

**Summary.—The dose–response relationships from the data described in Paper I were analysed. Among *unpromoted* animals, only doses sufficient to cause ulceration with subsequent promotion due to wound healing caused a rapid crop of tumours, so the dose–response curve exhibited strong *upward* curvature. Among *promoted* animals, the response of the skin to initiation appeared to have been nearly saturated by all DMBA doses tested, so that a 30-fold decrease in dose produced only a 3-fold decrease in effect. The dose–response relationship thus exhibited strong *downward* curvature.**

**Among promoted animals, estimation of the risks associated with very low doses of carcinogen by linear extrapolation through the origin from the effects of larger doses (which is often assumed to be conservative) would under-estimate the true risks by 10-fold or more. Our results emphasize that whereas linear interpolation from the results of high doses may be reasonable for data on the effects of continuous treatment with non-toxic dose levels of carcinogen, it may be misleading when extrapolating, as here, from the effects of single large doses.**

*The significance of downward curvature.*—The study of dose–response relationships is of interest for two reasons. First, the nature of the dose–response relationship may shed some light on the biological processes involved in carcinogenesis. For example, some hypotheses about cancer mechanisms predict an approximately linear dose response (i.e. proportionality between dose and number of tumours) while most others predict some upward curvature in the dose–response graph (*i.e.* that doubling the dose would more than double the effect). If *downward* curvature were demonstrated, this would exclude a large class of previously plausible hypotheses.

Second, because of the need to promulgate government regulations for various carcinogens, there is currently much dispute as to the magnitude of the risk likely to be associated with human exposure to lower doses of various carcinogens than can be studied in practicable experimental or epidemiological investigations. Some authors (*e.g.* Guess *et al.*, 1977; Crump *et al.*, 1976; Peto, 1979) have urged that a clear distinction be made between "one-shot" experiments (in which the effects of a single, usually toxic dose of carcinogen are studied) and "continuous" experiments (studying the effects of repeated doses, each insufficient separately or together to alter materially the cellular architecture of the target tissue). These authors have argued first, that even if no useful general rules emerge about the shapes of the dose–response relationships

* Requests for reprints to: Department of Pathology, University of Oulu, SF 90220, Oulu 22, Finland.

† Imperial Cancer Research Fund Reader in Cancer Studies, Nuffield Department of Clinical Medicine, Radcliffe Infirmary, Oxford OX2 6HE.

‡ Imperial Cancer Research Fund Cancer Epidemiology & Clinical Trials Unit, Oxford OX1 3QG.

in one-shot experiments, nevertheless there is generally likely to be either linearity or upward curvature in the relationship between cancer risk and dose rate in *continuous* experiments; and second, that for continuous experiments linear extrapolation downwards is generally therefore either correct or conservative, but should rarely if ever seriously *under*-estimate the risk of cancer under conditions of low-dose continuous carcinogenesis. Various other authors (*e.g.* Mantel & Schneiderman, 1975, or Cornfield, 1977) have, however, propounded arguments that linear extrapolation may almost always seriously *over*-estimate cancer risks at low doses, although these authors have not distinguished clearly between one-shot and continuous experiments. For a few carcinogens (particularly radiation) most of the animal and human data derives from one-shot studies (*i.e.* from animals or people exposed quickly to tens or hundreds of rads). Consequently, there is a need to determine whether the general rule of linearity or sublinearity (*i.e.* upward curvature) of dose–response relationships which is widely accepted for continuous carcinogenesis also applies to one-shot carcinogenesis. If it is incorrectly assumed to do so then human risks from low doses of such agents may be underestimated even by linear extrapolation, while any of the mathematical models (*e.g.* the "threshold", "virtually safe dose" or "probit" models) which assume upward curvature would lead to even more serious underestimates of risk. The present study suggests that this may on occasion be the case. (Note that, in this paper, "linear" extrapolation always means linear extrapolation towards zero *extra* risk at zero dose, *i.e.* proportionality of effect.)

### METHODS

Each of the 9 combinations of age at initiation and age at promotion described in the accompanying paper (Stenbäck *et al.*, 1981) was studied with initiating doses of 10, 30, 100 or 300 μg of DMBA. Consequently, within each group we may derive a dose-response relationship. Although any single such dose–response relationship may be somewhat unstable due to the limited number (40–80 mice) of mice at each of the 36 dose × time combinations studied, when the 9 dose–response relationships are averaged, a reliable and meaningful pattern should emerge. Since in many groups there was a substantial delay between initiation and promotion, we can examine two completely separate dose–response curves:

(a) the relationship between initiating dose and the response to promotion, when promotion does eventually occur, and

(b) the relationship between initiating dose and the number of tumours that arise soon after initiation in the absence of TPA promotion (due, perhaps, to the promotional effects of the healing of the ulcers and erosions caused by some DMBA doses).

In each case we may assess response by any 1 of 4 different indices of cancer incidence, *i.e.*

(i) the *total number of tumours* arising within a particular 20-week period of time (to which one mouse may contribute more than one tumour, which makes the calculation of reliable *P* values difficult), or

(ii–iv) the *number of tumour-bearing animals*, *i.e.* by a standard time-to-first-tumour analysis, with *P* values, of (ii) any tumour, irrespective of size or type, or (iii) 10mm tumours, or (iv) malignant tumours.

Details of the experimental and statistical methods are given in the accompanying paper by Stenbäck *et al.* (1981).

### RESULTS

### *The dependence on initiating dose of the effects of promotion*

No matter which index of response is adopted (small tumours, large tumours or malignant tumours), Fig. 1 shows clearly that the response to promotion exhibits striking downward curvature, the yield following initiation with only 10 μg being one-third of the yield with 300 μg, not one-thirtieth of it or less. (For numerical details, see Appendix Tables a and b.) In other words, estimation of the cancer yield from 10 μg by linear extrapolation downward from the yield at 300 μg would underestimate the true risk of the lower dose by a factor of about 10. If there were

FIG. 1.—Tumour response: O/E values giving dose–response relationships during promotion (from Appendix Tables a and b. ● All tumours within 20 weeks. ○ First tumours of any size or type (with approximate 95% confidence intervals). □ First 10mm tumours. △ First malignant tumour.

very marked genetic heterogeneity then this could produce a "shoulder" like those seen in Fig. 1 in the dose–response relationship for time to *first* tumour. However, if the intensity of effect is proportional to dose and the test animals do comprise a mixture of highly susceptible and very resistant genotypes, the affected animals might each have been expected to develop many tumours in response to the higher doses tested, and they did not. (Indeed there is at least as marked a shoulder in the O/E values for *total* tumours in Fig. 1 as in the O/E values for the numbers of tumour-bearing animals.) Moreover, even if we restrict our attention to the two lowest doses (10 μg and 30 μg) which do not suffice to cause much skin erosion or ulceration, we find that the yield from 10 μg exceeds one-third of the yield from 30 μg. It is as though even 10 μg suffices to initiate almost all the cells that are available to be initiated. It should be remembered that the doses given were administered in only 0·017 ml acetone, and so remained in an area probably less than 0·5 $cm^2$, so the lowest initiating stimulus we tested was well over 20 μg/$cm^2$, which is quite high. (To achieve this DMBA dose per unit area over the whole back of the mouse would require a dose of some hundreds of micrograms of DMBA in 0·2 ml of acetone.) The shape of the dose–response relationship at DMBA concentrations lower than the 20 or more μg/$cm^2$, which was the lowest dose we studied, cannot of course be inferred directly from our data, but presumably at some lower dose per unit area the response that will be elicited by promotion must start to decrease in proportion to the DMBA dose.

In Fig. 2 the life-table estimates (Pike & Roe, 1963; IARC, 1980) of the probabilities of tumourless animals remaining tumourless at various times after promotion are plotted, pooling all promoted animals irrespective of protocol. This is subject to some slight biases, as the proportions promoted at different times are different in the high- and low-dose groups, but it does illustrate adequately the lack of a 30-fold increase in the effects on initiation of increasing the dose from 10 μg to 300 μg of DMBA.

FIG. 2.—Relationship between dose of initiator and time to first tumour after start of promotion. Life-table estimate of never having had a tumour of any type. Data for the aggregate of all promoted animals (irrespective of ages at initiation or at promotion).

*The dependence of tumour yield on initiating dose at age 8 weeks in the absence of promotion with TPA*

Because, in many of our groups, there was a long interval between the single initiating dose of DMBA and subsequent promotion, we can use the observations made in these intervals to characterize the dose–response relationship for the tumorigenic effects of a single unpromoted dose of DMBA. The use of life-table techniques (Pike & Roe, 1963; IARC, 1980), treating animals as lost to observation when their TPA begins, allows us to pool the experience of several hundred animals in many different groups, and our life-table estimates of the tumorigenic effects of single doses of DMBA in 0·017 ml of acetone are therefore, initially at least, extremely reliable.

Fig. 3 illustrates the response, in terms of time to first tumour (of any size or type) to different single doses of DMBA administered at age 8 weeks. Although the pool of all groups of animals being observed after DMBA alone was depleted at 11, 18, 31, 51, and 71 weeks of age by mice being withdrawn in order to be promoted, there remained, at each DMBA dose level, over 200 mice at 31 weeks. In the absence of TPA no tumours arose in animals given 10 µg or 30 µg DMBA before Week 31, while a quarter to a third of the animals receiving 100 µg or 300 µg of DMBA developed tumours, presumably because of the promotional effects of the healing of DMBA-induced erosions and ulcers. This reproduces the findings of Turusov *et al.* (1971) that a single unpromoted DMBA dose of 200 µg in 0·033 ml of solvent caused severe necrosis with ulceration and scarring, with a quarter of the mice developing tumours within 32 weeks, but that doses of 100 µg or less in 0·033 ml of solvent did not cause severe necrosis nor any tumours within 6 months in the absence of promotion. (100 µg in 0·033 ml yields a concentration

FIG. 3.—Tumours without promotion. Life-table estimate of probability of never having had a tumour of any type. Data for aggregate of all animals initiated at age 8 weeks (treatment schedules b, c, d and e: animals scheduled to be promoted some time after they were initiated contribute to this graph only up to the time when they began to be promoted).

FIG. 4.—10mm tumours without promotion. Life-table estimate of probability of never having had a 10mm tumour. Data for aggregate of all animals initiated at age 8 weeks (treatment schedules b, c, d and e: animals scheduled to be promoted some time after they were initiated contribute to this graph only up to the time when they began to be promoted).

per unit area of skin intermediate between our doses of 100 μg and 30 μg in 0·017 ml). Likewise, Terracini *et al.* (1960) found that single unpromoted doses of 50, 100 and 200 μg DMBA in 0·05 ml acetone yielded ulcers in 0%, 25% and 75% of animals, and yielded tumours within 30 weeks in 0/22, 5/55 and 28/50 of the animals in these groups. These authors particularly noted that the tumours often arose from the ulcers or other less extreme skin lesions.

Many of the early tumours which we observed following DMBA alone regressed when the healing process was complete, and never became large. But, as time went by, quite substantial numbers of 10 mm tumours appeared (Fig. 4) among the unpromoted animals at the two higher doses, and many killed their hosts. By contrast, no 10mm tumours arose without promotion at the two lower dose levels (though 3 smaller malignancies did arise just before promotion cut observation of the animals short). Again, this finding reproduces the results of Terracini *et al.* (1960).

*The dependence on initiating dose at 48 weeks of tumour yield in the absence of promotion with TPA*

Among animals initiated at 48 weeks and not promoted for 23 weeks thereafter, there were somewhat more tumours at the top dose than at the top dose among animals initiated at 8 weeks. Surprisingly, however, the marked discontinuity in the dose–response relationship which Terracini *et al.* (1960), Turusov *et al.* (1971) and ourselves had found among animals initiated at 8 weeks was no longer evident, and even the lower two doses (10 and 30 μg DMBA) did elicit some tumours (not illustrated; numerical details in Appendix Table c). We do not know why no discontinuity is evident at 48 weeks. One obvious suggestion is that the likelihood of erosion or ulceration in response to a given DMBA dose may differ at 8 and at 48 weeks, or that the rate of (promotional) healing of such lesions may differ. Records of the apparent extent and rate of recovery of erosion or ulceration caused by unpromoted initiation at 48 weeks might have helped elucidate this point, but unfortunately such records are no longer available. In an independent replication of our 48-week study of unpromoted initiation (using animals of the same sex and strain, but from a different source), 200 mice were randomized, 50 to a group, between initiation with 10, 30, 100 or 300 μg DMBA in 0·017 ml acetone, and they were then observed for 50 more weeks. This more recent experiment reproduced, for initiation at 48 weeks, the dichotomous split between the two high doses and the two low doses, as seen in Figs 2, 3 and 4, and we therefore consider this, and not the response pattern in Appendix Table c, to be typical.

### DISCUSSION

*Initiation without promotion*

It is not surprising that, at least among animals initiated at 8 weeks of age, there is a very great difference in the immediate responses to 30 μg and to 100 μg of unpromoted initiation, for 30 μg in 0·017 ml of solvent rarely causes ulceration, whereas 100 μg in the same volume usually does so. Active healing processes undoubtedly have a promotional effect on mouse skin—for example, in this experiment it was often noticed that papillomas arose precisely out of the very thin line of epithelial cells overgrowing a DMBA-induced scar, and Terracini *et al.* (1960) report similar observations.

What is perhaps more interesting is the finding that the number of 10mm tumours arising 9–12 months after unpromoted initiation should exhibit the same discontinuity. This suggests that early promotion, either by wound healing or by TPA, can irreversibly "fix" certain cells (or microscopic clones of cells) in a higher risk category. In multistage terminology, this might be described as progression to a later "stage" of neoplastic development).

If there is indeed an irreversible cellular effect which can be produced by brief promotion, the simple dichotomy between normal cells and initiated cells will need to be extended to allow various categories of initiated cells (or microscopic clones of cells), and promotion itself may prove to be a multi-step process, as was suggested by Boutwell (1964) and Slaga *et al.* (1980).

*Initiation with promotion*

The downward curvature of the dose–response curve indicates that the generalizations about "conservative" risk estimation by linear extrapolation which have been proposed for continuous-carcinogenesis experiments cannot necessarily be carried over to single-dose experiments. This emphasizes the particular need to check the assumptions of linearity underlying the setting of safety levels for radiation exposure, since so much radiation data come from single-dose studies.

The reasons for such a dose–response relationship in the present context are unclear, especially since for another polyaromatic hydrocarbon, benzo(a)pyrene, Pereira *et al.* (1979) have shown simple proportionality between the dose applied to mouse skin and the amount subsequently bound to the epidermal DNA. One possible explanation is that even our lowest dose (20–50 $\mu g/cm^2$) sufficed to initiate nearly all the available target cells, and another is that higher doses actually kill off many of the initiated cells. Both are made plausible by other observations. Mondal & Heidelberger (1970) have shown *in vitro* that they can "transform" *most* of a population of cultured mammalian cells with MCA, and Kennedy *et al.* (1980) and Kennedy & Little (1980) have shown *in vitro* that they can "initiate" *most* of a population of cultured mammalian cells with an X-ray dose of 100 rad. Major & Mole (1978) have shown that if mice irradiated with 100 rad are randomized between getting and avoiding a further such dose of X-rays, those which get a further dose are less likely to develop leukaemia, presumably because the second dose kills more preleukaemic cells than it generates. Finally, returning to DMBA on mouse skin, it has been shown that 100 doses of 1 $\mu g$ of DMBA, given twice weekly are much more tumorigenic than a single dose of 100 $\mu g$ (Saffiotti & Shubik, 1956), which again is what would be expected if small doses suffice to initiate almost all the cells which are temporarily at risk of initiation.

In retrospect, since we believe that we did saturate the possible response of the mouse skin, we do not recommend that future workers treat such a small area of the back of the mouse as we did. Using a volume of solvent sufficient to spread over the whole shaved back, at dose levels per unit area which are sufficiently low not to saturate the possible response of the skin,

(i) will avoid any complication due to promotional wound healing;

(ii) will allow any factors modifying the efficacy of DMBA to be measured; and

(iii) will cause more target cells to be exposed, which will improve the statistical accuracy of the results.

If such doses do not produce high enough tumour yields, it would perhaps be better to study initiation by a few weekly very low doses rather than by single substantial doses.

REFERENCES

BOUTWELL, R. K. (1964) Some biological aspects of skin carcinogenesis. *Progr. Exp. Tumor Res.*, **4**, 207.

CORNFIELD, J. (1977) Carcinogenic risk assessment. *Science*, **198**, 693.

CRUMP, K. S., HOEL, D. G., LANGLEY, C. H. & PETO, R. (1976) Fundamental carcinogenic processes and their implications for low dose risk assessment. *Cancer Res.*, **36**, 2973.

GUESS, H., CRUMP, K. S. & PETO, R. (1977) Uncertainty estimates for low-dose-rate extrapolations of animal carcinogenicity data. *Cancer Res.*, **37**, 3475.

INTERNATIONAL AGENCY FOR RESEARCH ON CANCER (1980) Guidelines for simple, sensitive significance tests for carcinogenic effects in long-term animal experiments. In *IARC Monographs on the Evaluation of the Carcinogenic Risk of Chemicals to Humans*, Suppl. 2. Ed. Montesano. Lyon: I.A.R.C. p. 311.

KENNEDY, A. R., FOX, M., MURPHY, G. & LITTLE, J. B. (1980) Relationship between X-ray exposure and malignant transformation in C3H 10T½ cells. *Proc. Natl Acad. Sci., U.S.A.*, **77**, 7262.

KENNEDY, A. R. & LITTLE, J. B. (1980) An investigation of the mechanism for the enhancement of radiation transformation *in vitro* by TPA. *Carcinogenesis*, **12**, 1039.

MAJOR, I. R. & MOLE, R. H. (1978) Myeloid leukaemia in X-ray irradiated CBA mice. *Nature*, **272**, 455.

MANTEL, N. & SCHNEIDERMAN, M. A. (1975) Estimating "safe" levels: A hazardous undertaking. *Cancer Res.*, **35**, 1379.

MONDAL, S. & HEIDELBERGER, C. (1970) *In vitro* malignant transformation by methylcholanthrene of the progeny of single cells from C3H mouse prostate. *Proc. Natl Acad. Sci., U.S.A.*, **65**, 219.

PEREIRA, M. A., BURNS, F. J. & ALBERT, R. E. (1979) Dose response for benzo(a)pyrene adducts in mouse epidermal DNA. *Cancer Res.*, **39**, 2556.

PETO, R. (1979) Detection of risk of cancer to man. *Proc. R. Soc. Lond. B.*, **205**, 111.

PIKE, M. C. & ROE, F. J. C. (1963) An acturial method of analysis of an experiment in two-stage carcinogenesis. *Br. J. Cancer*, **17**, 605.

SAFFIOTTI, V. & SHUBIK, P. (1956) The effects of low concentrations of carcinogen in epidermal carcinogenesis: A comprison with promoting agents. *J. Natl Cancer Inst.*, **16**, 961.

SLAGA, T. J., KLEIN-SZANTO, A. J. P., FISCHER, S. M., NELSON, K. & MAJOR, S. (1980) Studies on the mechanism of action of anti-tumor promoting agents: Their specificity in two-stage promotion. *Proc. Natl Acad. Sci., U.S.A.*, **77**, 2251.

STENBÄCK, F., PETO, R. & SHUBIK, P. (1981) Initiation and promotion at different ages and doses in 2200 mice. I. Methods, and the apparent persistence of initiated cells. *Br. J. Cancer*, **44**, 1.

TERRACINI, B., SHUBIK, P. & DELLA PORTA, G. (1960) A study of skin carcinogenesis in the mouse with single applications of 9:10-dimethyl-1:2-benzanthracene at different dosages. *Cancer Research*, **20**, 1538.

TURUSOV, V., DAY, N. E., ANDRIANOV, L. & JAIN, D. (1971) Influence of dose on skin tumors induced in mice by single application of 7,12-dimethylbenz(a)anthracene. *J. Natl Cancer Inst.*, **47**, 105.

### Appendix

TABLE C.—*Numbers of tumours arising without TPA promotion within 23 weeks of initiation for animals initiated at 8 or 48 weeks of age*

| Age at initiation | 300 μg initiation | | 100 μg initiation | | 30 μg initiation | | 10 μg initiation | |
|---|---|---|---|---|---|---|---|---|
| | O | MS | O | MS | O | MS | O | MS |
| 8 weeks | 94 | 221 | 66 | 211 | 0 | 210 | 0 | 214 |
| | 0·43 T/M | | 0·31 T/M | | 0·00 T/M | | 0·00 T/M | |
| 48 weeks | 46 | 55 | 10 | 59 | 6 | 63 | 2 | 72 |
| | 0·84 T/M | | 0·17 T/M | | 0·10 T/M | | 0·03 T/M | |

MS = Number of Mice Surviving at least 23 weeks after initiation.

O = Number of tumours, irrespective of size or type, Observed to arise on these survivors during the 23 weeks following initiation.

T/M = Tumours per mouse = O/MS.